# Prognostic Value of Systemic Immune Inflammation Index in Squamous Cell Lung Cancer

**DOI:** 10.3390/jcm14072219

**Published:** 2025-03-25

**Authors:** Sefika Umihanic, Lora Novakovic, Lejla Alidzanovic, Medina Bandovic Kuduzovic, Anida Sehic, Almedina Muhic, Amila Kovcic, Nejra Selak

**Affiliations:** 1 Clinic for Oncology and Radiotherapy, University Clinical Center Tuzla, 75000 Tuzla, Bosnia and Herzegovina; umihanics@gmail.com (S.U.); lejla.alidzanovic@gmail.com (L.A.); anida.sehic@gmail.com (A.S.); almedina_terzic@hotmail.com (A.M.); kovcic.amila1@gmail.com (A.K.); 2 Clinic for Pulmology, University Clinical Center Banja Luka, 78000 Banja Luka, Bosnia and Herzegovina; lora.novakovic@kc-bl.com; 3 Clinic for Pulmology, University Clinical Center Tuzla, 75000 Tuzla, Bosnia and Herzegovina; medinakud@gmail.com; 4 Pathology Department, University Clinical Center Tuzla, 75000 Tuzla, Bosnia and Herzegovina

**Keywords:** squamous cell lung cancer, overall survival, systemic immune-inflammation index, neutrophil-to-lymphocyte ratio, platelet-to-lymphocyte ratio

## Abstract

**Background/Objectives:** Squamous cell lung cancer (SCC) presents a significant treatment challenge due to its poor prognosis and limited therapeutic options. In many resource-limited countries, access to advanced molecular testing is often unavailable, making the identification of novel and reliable prognostic markers crucial for improving patient selection for systemic treatments. **Methods:** This single-center, retrospective study investigated the prognostic value of inflammatory biomarkers, including the systemic immune-inflammation index (SII), neutrophil-to-lymphocyte ratio (NLR), and platelet-to-lymphocyte ratio (PLR), in 134 patients diagnosed with SCC. Patients were stratified into groups based on optimal cut-off values determined by ROC analysis for each biomarker. **Results:** Elevated levels of the SII, NLR, and PLR were significantly associated with shorter overall survival in patients with SCC (all *p* < 0.05). **Conclusions:** These easily accessible and cost-effective laboratory parameters are particularly valuable in settings where molecular testing is not available, aiding in the identification of high-risk patients and optimizing treatment selection for chemotherapy.

## 1. Introduction

Squamous cell lung cancer (SCC) accounts for approximately 30% of all non-small cell lung cancers (NSCLCs) [1]. SCC primarily affects smokers and has high mutation rates and complex genomic alterations. In contrast to lung adenocarcinoma, where over 40% of cases have actionable targets, identifying driver mutations and actionable targets in SCC has proven to be challenging [2]. The NCCN guidelines for NSCLCs recommend broad, panel-based next-generation sequencing for identifying actionable targets in NSCLCs, including mutations in EGFR, ALK, and PD-L1 [1]. However, in developing countries, such testing and immunotherapies are often inaccessible, leaving chemotherapy as the primary treatment option for SCC patients. The high cost and unavailability of molecular testing further limit its routine use in these regions [3].

Inflammation is now considered a key enabler of tumorigenesis, promoting multiple cancer hallmarks [4]. Initially considered a defense mechanism, inflammatory responses paradoxically enhance tumor progression by supplying bioactive molecules that sustain proliferation, inhibit cell death, and support angiogenesis, invasion, and metastasis. Inflammatory cells—including macrophages, neutrophils, mast cells, and lymphocytes—release cytokines, chemokines, and proteases that amplify these effects, fostering tumor growth and progression [5]. Additionally, reactive oxygen species generated by inflammatory cells drive genetic mutations, accelerating cancer evolution. Tumor-infiltrating immune cells further sustain angiogenesis, facilitate invasion, and aid metastatic dissemination, making inflammation a crucial factor in cancer progression.

Regarding circulating immune cells, the tumor-derived secretome can affect distant sites like the bone marrow and spleen, promoting myelopoiesis [6]. While myeloid cells have been known to contribute to tumor progression for over a century, their role in angiogenesis, cell invasion, and metastasis has only been recognized in the past two decades. Cancer myelopoiesis leads to the accumulation of immature myeloid cells, like myeloid-derived suppressor cells (MDSCs), in circulation, unlike the rapid differentiation seen in acute infections. Circulating granulocytes, especially neutrophils, are increased in cancer. Neutrophils, usually released only when mature, may also be released as precursors during inflammation.

Platelets have recently been proven as a significant predictor of disease severity and patient outcomes across COVID-19, sepsis, and systemic lupus [7] and play a crucial role in oncology. In cancer progression, they protect circulating tumor cells from immune attacks, promote metastasis through tumor cell-induced platelet aggregation, facilitate immune evasion, interact with neutrophils to enhance tumor-associated thrombosis, and induce epithelial–mesenchymal transition, all of which contribute to increased tumor invasiveness and poorer prognosis. The involvement of platelets in both cancer and inflammatory conditions like COVID-19 highlights their broader role in disease pathophysiology, making them a key target for therapeutic strategies [8].

Inflammatory biomarkers, such as the neutrophil-to-lymphocyte ratio (NLR) and platelet-to-lymphocyte ratio (PLR), have been proposed as novel indicators of poor prognosis in NSCLC [9,10,11]. The systemic immune-inflammation index (SII) is a novel marker that reflects the relationship between inflammatory and immunogenic components of the tumor and represents a valid prognostic marker in different types of malignancies [12], including NSCLC [13]. While most studies examine these markers in the context of NSCLC as a whole, our research specifically targeted SCC

The aim of this study was to evaluate the prognostic significance of the SII in patients with lung SCC and to compare its prognostic value with established inflammatory biomarkers such as NLR and PLR. This evaluation is particularly pertinent in the context of developing countries where advanced molecular testing and targeted therapies are often unavailable.

## 2. Materials and Methods

### 2.1. Patients

The study included 134 patients diagnosed with squamous cell lung cancer between January 2020 and August 2022. The inclusion criteria were as follows: (i) a confirmed diagnosis of primary SCC by pathologic examination (histology and/or cytology), classified according to the seventh edition of the TNM classification for NSCLC, (ii) diagnosis of stage IIIB or IV SCC; patients with earlier-stage SCC were included only if surgical intervention was not feasible, and (iii) no prior chemotherapy or radiotherapy before blood sample collection. The exclusion criteria were (i) a history of other malignancies and (ii) previous treatment modalities for lung SCC.

### 2.2. Collection of Clinical Data

Demographic and clinical data were recorded for each patient upon hospital admission. Variables included age, sex, smoking status, time to disease progression, and overall survival (OS). Progression-free survival (PFS) was defined as the time from the start of chemotherapy to disease progression or death from any cause before disease progression. OS was defined as the time from the start of chemotherapy to death from any cause or the last follow-up. Blood samples were collected either at the diagnosis or prior to chemotherapy and hematologic parameters were retrieved retrospectively from electronic medical records.

### 2.3. Neutrophil-to-Lymphocyte Ratio (NLR), Platelet-to-Lymphocyte Ratio (PLR), and Systemic Immune-Inflammation Index (SII) Evaluation

The NLR was defined as the ratio of the absolute number of neutrophils to the absolute number of lymphocytes. The PLR was determined as the ratio of the absolute number of platelets to the absolute number of lymphocytes. The SII was calculated using the following formula: platelet count × neutrophil count/lymphocyte count [9]. To determine the optimal cut-off values for the SII, NLR, and PLR, receiver operating characteristic (ROC) curve analyses were performed separately. The obtained optimum cut-off points for the SII, NLR, and PLR were 672.9, 2.564, and 155.1, respectively.

### 2.4. Statistical Analysis

SPSS (version 27, IBM Corp) and MedCalc (version 20, MedCalc Software Ltd., Ostend, Belgium) software were used for all statistical data processing. Cut-off values were primarily determined by Youden’s index and validated with X-Tile software (version 3.0, Yale School of Medicine, New Haven, CT, USA), through Kaplan–Meier survival curves. ROC curve analysis was conducted to optimize sensitivity and specificity. The log-rank test was performed to compare survival distributions between groups. For further statistical analyses, the Cox regression model and non-parametric tests (Kruskal–Wallis and median test) were applied. In the Cox regression models, we assessed several variables, including age, sex, smoking history, and inflammatory indices (the SII, NLR, and PLR).

## 3. Results

The clinical characteristics of patients are shown in Table 1. In total, 134 patients were analyzed, of whom 120 were men (89.55%) and 14 were women (10.45%). Most of the patients had a history of smoking (110; 82.09%). The mean age was 69 years (±6.83). An analysis of the complete blood count performed at the time of diagnosis or before the start of chemotherapy showed median values for the SII, NLR, and PLR at 981.73, 3.22, and 172.0, respectively. The average survival time was 12.01 months (±9.17).

The normal reference ranges for the hematological parameters measured are as follows: lymphocytes (1.0–3.0 × 10⁹/L), monocytes (0.2–1.0 × 10⁹/L), neutrophils (2.0–7.0 × 10⁹/L), and thrombocytes (150–410 × 10⁹/L) [14].

No statistically significant differences were observed in progression or survival based on sex or smoking status. However, a significant difference was found based on disease stage.

There were statistically significant differences in monocyte, neutrophil, and thrombocyte counts, as well as the SII, between patients who survived and those who did not. The PLR also showed a statistically significant difference according to disease stage (Table 2).

### 3.1. ROC Analysis

**Figure 1:**
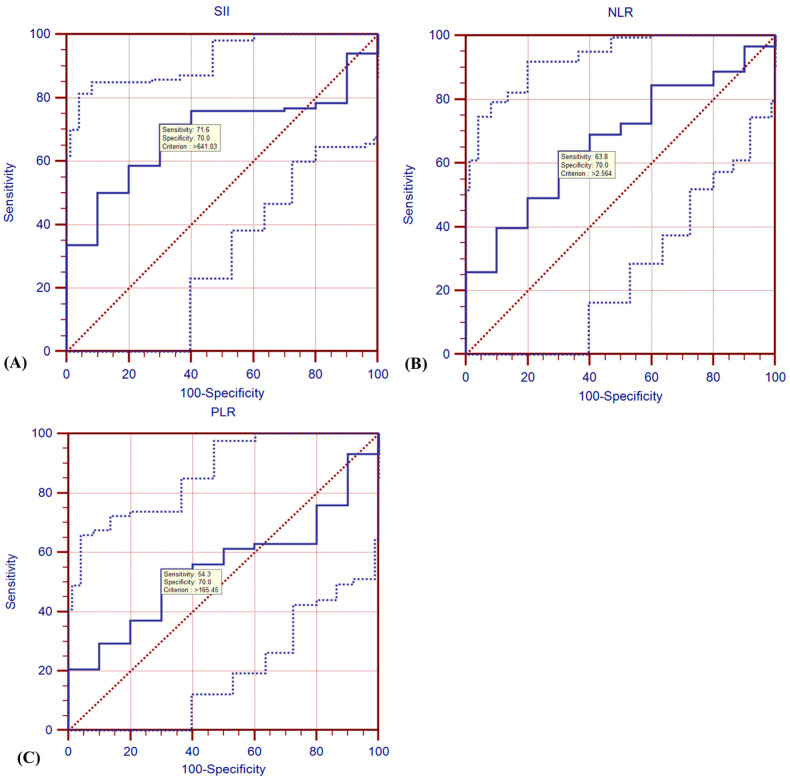
TheROC curves for (**A**) SII (**B**) NLR, and (**C**) PLR in lung SCC patients. The solid blue line represents the ROC curve, while the dashed blue lines indicate the confidence intervals. The red diagonal line represents the reference line (chance level). The sensitivity and specificity values at the optimal cutoff point are marked within each graph. (**A**) The AUC for the SII is 0.690 and the cut-off value is 641.03, with a sensitivity of 0.716 and specificity of 0.700; (**B**) the AUC for NLR is 0.674 and the cut-off value is 2.564, with a sensitivity of 0.638 and specificity of 0.700; (**C**) the AUC for PLR is 0.553, and the cut-off value is 165.45, with a sensitivity of 0.643 and specificity of 0.700. PLR: platelet-to-lymphocyte ratio, NLR: neutrophil-to-lymphocyte ratio, SII: systemic immune-inflammation index, ROC: receiver operating characteristics, AUC: area under the curve.

To determine the prognostic value of the SII, NLR, and PLR, we performed ROC curve analysis to establish the optimal cut-off values for the SII, NLR, and PLR and evaluated their prognostic ability to predict survival. The cut-off values, along with their respective sensitivity, specificity, and *p*-values were as follows: SII—641.03 cut-off value, 71.6% sensitivity, 70.0% specificity, and *p* = 0.0028; NLR—2.564 cut-off value, 63.8% sensitivity, 70.0%, specificity, and *p* = 0.02; and PLR—165.45 cut-off value, 54.3% sensitivity, 70.0% specificity, and *p* = 0.482. The SII and NLR demonstrated prognostic significance, while the PLR did not (Figure 1). Both the SII and NLR demonstrated significant prognostic value, with higher values correlating with poorer survival outcomes. In contrast, the PLR did not reach statistical significance, suggesting it may not be a reliable predictor in this setting.

### 3.2. Cox Regression Model

**Figure 2:**
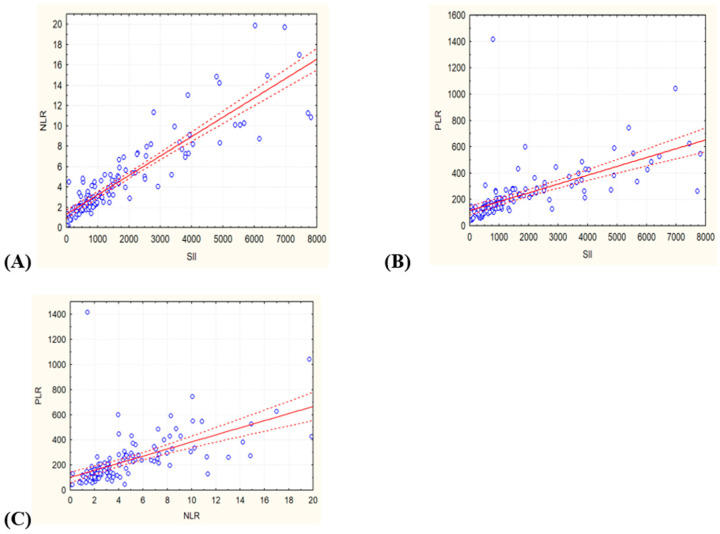
Correlation between the variables: (**A**) SII and NLR (r = 0.899), (**B**) SII and PLR (r = 0.648), and (**C**) NLR and PLR (r = 0.577). The solid red line represents the least squares regression line, while the dashed red lines indicate the 95% confidence interval of the regression. PLR: platelet-to-lymphocyte ratio, NLR: neutrophil-to-lymphocyte ratio, SII: systemic immune-inflammation index.

Using a Cox regression model, we found significant prognostic values for all variables for overall survival (Table 3). However, when all three variables were included in a combined model, none remained statistically significant. A correlation analysis among the SII, NLR, and PLR was performed to assess the degree of interrelationship between these inflammatory markers and to understand whether they provide overlapping or distinct prognostic information in SCC. It showed strong relationships between the SII and NLR (r = 0.899) and a moderate positive correlation between the SII and PLR (r = 0.648) and the NLR and PLR (r = 0.577) (Figure 2).

As the inflammatory indices were not significant in the multivariate model, we also performed an additional multivariate Cox regression analysis including only one inflammatory index at a time to assess whether including the variables “smoking history”, “sex”, and “age” would increase the accuracy of the prediction model. Only positive smoking history significantly enhanced the model’s accuracy (*p* = 0.04) (Table 4).

### 3.3. Kaplan–Meier Survival Curves

**Figure 3:**
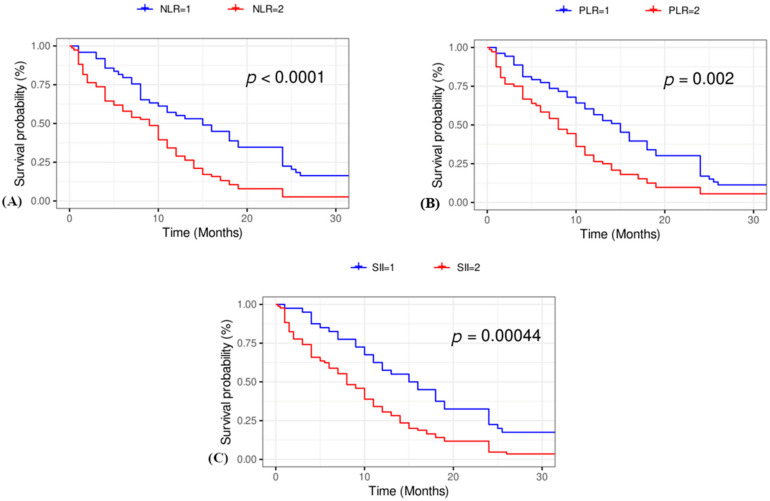
Kaplan–Meier survival curves for patients stratified by NLR, PLR, and SII scores. (**A**) NLR score. Patients with a NLR = 1 (<2.564) are represented by the blue curve, and the red curve represents those with a NLR = 2 (>2.564) (log rank test *p* < 0.0001). (**B**) PLR score. Patients with a PLR = 1 (<165.45) are represented by the blue curve, and the red curve represents those with a PLR = 2 (>165.45) (log-rank test *p* = 0.002). (**C**) SII score. Patients with a SII = 1 (<631.03) are represented by the blue curve, and the red curve represents those with a SII = 2 (>641.03) (log-rank test *p* = 0.00044). PLR: platelet-to-lymphocyte ratio, NLR: neutrophil-to-lymphocyte ratio, SII: systemic immune-inflammation index.

Kaplan–Meier curves suggested that elevated NLR, PLR, and SII values were associated with worse survival outcomes in SCC patients (Figure 3). Among these indices, the SII demonstrated the highest prognostic value.

## 4. Discussion

We evaluated the prognostic significance of inflammatory indices in the progression and survival of patients with lung SCC. Our finding revealed that SII values were significantly higher in patients with a shorter survival time. Although only the SII was significant for overall survival in the Kruskal–Wallis test, all three indices (SII, NLR, and PLR) were significant in univariate Cox regression. Kaplan–Meier survival analysis revealed that the elevated NLR, PLR, and SII were significantly associated with worse overall survival in SCC patients. The identification of optimal cut-off values for the SII and NLR allows for the stratification of patients into high- and low-risk groups, which may aid in prognostic assessment and treatment decision making in SCC patients, consistent with findings from other studies [13,15].

The correlation analysis showed a very strong correlation between the SII and NLR (r = 0.899), suggesting that these indices largely reflect the same inflammatory processes, primarily driven by neutrophil and lymphocyte counts. This is important for prognostic modeling, as including both in multivariate analyses may lead to multicollinearity, potentially distorting effect estimates. In contrast, the PLR showed weaker correlations with the SII (r = 0.648) and NLR (r = 0.577), suggesting that it may capture different inflammatory dynamics, possibly related to platelet-driven processes. These findings are relevant to SCC prognosis, as they help refine the selection of inflammatory markers for survival prediction and ensure robust statistical modeling in further analyses.

In resource-limited settings, where the availability of immunotherapy for SCC patients is often insufficient, standard chemotherapy remains the primary treatment option despite its broad toxicity profile and associated side effects. Given these challenges, identifying reliable prognostic factors is crucial for optimizing treatment strategies with cytotoxic therapy. These indices are particularly valuable due to their accessibility, quick detection, and low cost, making them essential tools in the management of SCC.

SII is associated with cancer prognosis through the roles of neutrophils, platelets, and lymphocytes. Neutrophils contribute to the tumor microenvironment and may support tumor growth and metastasis, while platelets can protect tumor cells from immune attacks and promote their spread. Lymphocytes, on the other hand, are involved in tumor cell elimination through immune responses. Elevated SII levels may reflect an imbalance in these immune processes, potentially indicating more aggressive tumor behavior and poorer outcomes in cancer patients [16]. In SCC patients, a lower NLR was associated with longer recurrence-free survival after surgery. Additionally, the NLR was inversely related to the presence of T cells and B cells in tumor tissues, suggesting that the NLR could be a valuable predictor of postoperative recurrence and may provide insights into the immune landscape of the tumor microenvironment [17]. Combined chemotherapy and immunotherapy are now standard treatments for lung cancer, alongside anti-PD-1/PD-L1 antibody therapy [1]. The NLR showed promise as a predictor of immune checkpoint blockage efficacy in NSCLC patients [18,19].

A large study analyzing 6101 NSCLCpatients, including 1852 with SCC, identified tumor stage at diagnosis and performance status as the most significant independent prognostic factors for five-year survival across all histological types [20]. Additionally, weight loss before diagnosis was linked to poorer outcomes across all NSCLC subtypes. Among SCC-specific factors, smoking history (measured in pack-years) was found to be an independent negative prognostic marker, highlighting the impact of tobacco exposure on survival in these patients. The TNM stage remains the primary predictive factor for adjuvant treatment decisions in clinical practice. While clinical trials on postoperative chemotherapy are increasing, outcomes remain modest, and no prognostic or predictive biomarkers have been established for routine patient management [21]. Combining inflammatory indices with other markers further enhances their utility, such as nutrition-based scores [22]. Inflammatory indices like the NLR and SII are increasingly integrated into prognostic scores across cancers. In esophageal squamous cell carcinoma, the F-NLR score improves prognostic accuracy over traditional markers [23], while in gastric the NLR-CA19-9 score enhances survival prediction [24].

If the prognostic significance of the SII is confirmed in larger, multi-center prospective studies, preoperative or pretreatment SII levels could serve as a valuable tool for risk stratification and personalized treatment planning [25]. For patients with high preoperative SII values, intensified surveillance strategies or adjuvant therapy may be warranted to mitigate poor outcomes. Conversely, in select cases, aggressive interventions such as surgery, radiotherapy, or systemic therapies (chemotherapy, targeted therapy, immunotherapy) could be reconsidered to balance therapeutic benefits against potential adverse effects. Additionally, integrating anti-inflammatory treatments into treatment protocols may offer a novel approach to improving outcomes in patients with elevated SII values. Future research should focus on validating these findings in diverse patient populations and exploring the impact of SII-guided treatment modifications on long-term survival and disease progression.

This study has several limitations. It is a retrospective, single-center study with a small sample size which limits its generalizability. The inflammatory indices were measured at one time point, and their variation over time and under different conditions was not analyzed. This limited our ability to evaluate their dynamic changes over time or in response to treatment. As such, the study may not fully reflect the long-term prognostic value of these indices or their potential changes in response to therapeutic interventions. Also, we lacked data on the molecular profiles of the patients. Regarding the study period (2020–2022) which overlapped with the COVID-19 pandemic, there could have been potential confounding effects of COVID-19 on inflammatory markers such as the SII, NLR, and PLR. COVID-19 infection has been shown to alter immune responses and inflammatory profiles [26], which may have influenced the inflammatory indices of our patients. However, we did not collect data on patients’ COVID-19 status or infection history during the study period, and as such, we cannot fully account for its potential impact. Future studies should incorporate information on COVID-19 status to better assess its possible confounding effects on inflammatory biomarkers and ensure more accurate analyses.

## 5. Conclusions

Given the practicality, low cost, and quick detection of systemic inflammatory markers, their use in routine clinical practice is essential. Our findings show elevated inflammatory indices in patients with lung SCC, confirming a poor prognosis related to disease control and treatment limitations. While the exploration of molecular markers remains crucial, our findings highlight the importance of further investigating combinations of inflammatory markers to enhance their utility in predicting outcomes and tailoring treatment and follow-up strategies in lung SCC patients.

## Figures and Tables

**Table 1:** Clinical characteristics of patients.

Variable	
Age, mean	69.10 ± 6.83
Sex	
Male	120 (89.55%)
Female	14 (10.45%)
Smoking history	
No	24 (17.91%)
Yes	110 (82.09%)
Stage	
I	3 (2.24%)
II	15 (11.19%)
III	65 (48.51%)
IV	51 (38.06%)
Progression	
No	27 (20.61%)
Yes	104 (79.39%)
Survival	
Yes	11 (8.40%)
No	120 (91.60%)
Lymphocytes, mean (×10⁹/L)	1.80 ± 0.92
Monocytes, mean (×10⁹/L)	0.71 ± 0.38
Neutrophils, mean (×10⁹/L)	6.82 ± 4.63
Thrombocytes, mean (×10⁹/L)	333.25 ± 146.07
Time to progression, months, mean	9.80 ± 8.86
Overall survival, months, mean	12.01 ± 9.17
SII, median	981.72 (522.91–2276.86)
NLR, median	3.22 (2.12–5.89)
PLR, median	172.00 (124.17–279.34)

**Table 2:** Comparison of inflammatory indices across variables.

Variable		Lymphocytes (Mean) * (×10⁹/L)	*p* Value	Monocytes (Mean) * (×10⁹/L)	*p* Value	Neutrophils (Mean) * (×10⁹/L)	*p* Value	Thrombocytes (Mean) * (×10⁹/L)	*p* Value	SII (Median) #	*p* Value	NLR (Median)#	*p* Value	PLR (Median) #	*p* Value
**Sex**	Male	1.80 ± 0.93	0.96	0.72 ± 0.04	0.54	6.74 ± 4.75	0.62	327.56 ± 143.61	0.19	908.26 (522.91–2204.26)	0.31	3.16 (2.12–5.89)	0.45	166.96 (127.17–276.32)	0.41
	Female	1.79 ± 0.90		0.65 ± 0.12		7.39 ± 3.51		380.79 ± 163.08		1479.92 (576.50–2517.50		4.03 (2.85–5.07)		212.07 (131.32–346.84)	
**Smoking history**	Yes	1.85 ± 0.95	0.24	0.72 ± 0.04	0.75	7.01 ± 4.82	0.33	332.03 ± 150.12	0.84	1070.44 (522.67–2262.50)	0.97	3.25 (2.14–5.89)	0.56	171.01 (118.29–276.32)	0.33
	No	1.58 ± 0.78		0.69 ± 0.07		5.99 ± 3.62		338.71 ± 129.20		864.25 (598.97–2392.22)		2.69 (1.90–6.18)		197.13 (135.02–313.05)	
**Stage**	I	1.72 ± 0.21	0.215	NA	0.15	5.04 ± 2.65	0.51	240.33 ± 38.42 ^b^	0.52	641.03 (506.29–813.21	0.098	2.56 (1.86–4.11)	0.54	160.26 (101.02–165.45)	**0.009**
	II	2.09 ± 0.83		0.64 ^a^		6.20 ± 3.51		299.36 ± 140.83		637.27 (446.40–1383.71)		2.24 (1.99–3.97)		137.06 (105.23–176.37)	
	III	1.90 ± 0.71		0.68 ± 0.38		6.44 ± 4.66		337.42 ± 135.61		908.26 (576.50–1677.72)		2.81 (2.05–4.92)		171.01 (118.29–262.84)	
	IV	1.59 ± 1.16		0.75 ± 0.40S		7.55 ± 4.89		342.98 ± 163.28		1697.06 (535.38–3619.08)		4.80 (2.85–8.30)		240.99 (138.67–372.57)	
**Progression**	Yes	1.77 ± 0.39	0.56	0.73 ± 0.04	0.36	7.05 ± 4.91	0.37	337.48 ± 150.04	0.53	1004.17 (568.70–2517.50)	0.29	3.25 (2.17–5.89)	0.22	195.83 (128.67–280.77)	0.19
	No	1.94 ± 0.88		0.63 ± 0.12		6.13 ± 3.33		317.77 ± 134.99		751.20 (458.50–1349.47)		2.56 (2.03–4.50)		153.31 (113.33–237.74)	
**Survival**	No	1.81 ± 0.94	0.69	0.74 ± 0.37	**0.002**	7.12 ± 4.72	**0.04**	340.60 ± 150.13	0.07	1070.39 (572.60–2532.01)	**0.046**	3.24 (2.14–6.92)	0.068	179.28 (123.22–280.06)	0.575
	Yes	1.69 ± 0.66		0.21 ± 0.16		4.00 ± 1.89		257.73 ± 72.61		529.36 (506.29–878.94)		2.30 (1.86–3.35)		154.89 (141.33–237.74)	

Bold indicates statistical significance; ^a^ only one patient, ^b^ only 3 patients; * *t*-test or ANOVA; ^#^ Kruskall Wallis test; NA: no data available. The normal reference ranges for the hematological parameters measured are as follows: lymphocytes (1.0–3.0 × 10⁹/L), monocytes (0.2–1.0 × 10⁹/L), neutrophils (2.0–7.0 × 10⁹/L), and thrombocytes (150–410 × 10⁹/L) [14].

**Table 3:** Univariate and multivariate Cox regression analysis of overall survival of patients.

		Univariate Cox Regression		Multivariate Cox Regression	
		HR (95% CI)	*p*-value	HR (95% CI)	*p*-value
Age		1.02 (0.99–1.05)	0.183		
Smoking history	No	1			
	Yes	1.18 (0.74–1.86)	0.487		
Sex	Male	1			
	Female	1.25 (0.70–2.22)	0.451		
PLR	1	1			
	2	1.23 (1.06–1.44)	0.007	1.19 (0.73–1.95)	0.478
NLR	1	1			
	2	1.38 (1.12–1.71)	0.003	1.54 (0.90–2.62)	0.113
SII	1	1			
	2	1.39 (1.09–1.77)	0.001	1.53 (0.81–2.88)	0.192

PLR: platelet-to-lymphocyte ratio, NLR: neutrophil-to-lymphocyte ratio, SII: systemic immune-inflammation index: NLR = 1 (<2.564); NLR = 2 (>2.564); PLR = 1 (<165.45); PLR = 2 (>165.45); SII = 1 (<641.03); SII = 2 (>641.03).

**Table 4:** Multivariate Cox regression analyses of the overall survival of patients.

Model 1	HR	95% CI	*p* Value
SII	1.449	1.134–1.851	0.003
Age	1.008	0.981–1.036	0.546
Smoking history (Ref: No)	0.698	0.439–1.111	0.13
Sex (Ref: Male)	0.687	0.382–1.234	0.209
**Model 2**			
NLR	1.439	1.159–1.788	0.001
Age	1.005	0.978–1.032	0.73
Smoking history (Ref: No)	0.663	0.414–1.063	0.088
Sex (Ref: Male)	0.726	0.405–1.303	0.283
**Model 3**			
PLR	1.335	1.13–1.578	0.001
Age	1.005	0.978–1.032	0.733
Smoking history (Ref: No)	0.59	0.357–0.976	0.04
Sex (Ref: Male)	0.679	0.378–1.22	0.196

## Data Availability

Data will be made available by the corresponding author upon reasonable request.

## Abbreviations

The following abbreviations are used in this manuscript:

SII	Systemic immune-inflammation index
NLR	Neutrophil-to-lymphocyte ratio
PLR	Platelet-to-lymphocyte ratio
ROC	Receiver operating characteristic
SCC	Squamous cell carcinoma
NSCLC	Non-small cell lung cancer
ICB	Immune checkpoint blockade
PD-1	Programmed cell death protein 1
PD-L1	Programmed death-ligand 1
SCCA	Squamous cell carcinoma antigen
